# Supplementary material for: Full-scale evaluation of methane production under oxic conditions in a mesotrophic lake
Source: Nat Commun. 2017 Nov 21;8:1661. doi: 10.1038/s41467-017-01648-4 (PMC5698424; doi:10.1038/s41467-017-01648-4)
Supplement: Supplementary file 1 — Supplementary Information [file 41467_2017_1648_MOESM1_ESM.pdf]

Supplementary Note 1

In 1898, the first signs of an incipient eutrophication occurred in Lake Hallwil with the appearance of the "Burgundy algae" *Planktothrix rubescens* followed by oxygen depletion in the hypolimnion and decreasing fish catch, leading progressively by the mid 1970's to a maximum phosphorous (P) load of 16 T yr^{-1} (mean total P of 250 mg m^{-3})[1,2]. First restoration measures aimed to reduce nutrient load through sewage diversion and treatment, followed later by measures to limit nutrient loss from agriculture. Consequently, the P load continuously decreased until todays 3 T yr^{-1} [3]. The re-oligotrophication process was supported since 1986 by the installation of a bubble-plume hypolimnetic aeration system using pure oxygen designed to prevent a complete loss of oxygen in the deep water [4,5]. The so called "Tanytarsus" system, placed on the lake bed at around 46 m depth, is made of six diffusers of 6.5 m in diameter, positioned in a circular configuration of 200 m diameter. The gas flow rate of the system is regulated such that, while preventing anoxic conditions in the deep water, the rising bubble plume does not affect the stratification of the water column in summer[6]. In winter Lake Hallwil water column has been artificially mixed with course bubbles using compressed air. Given the significant improvement of lake water quality[7], aeration measures were largely reduced in the last years. While needing 2.5 to 4 tons of oxygen per day from May to October in the 1990's to prevent local anoxia, less than 1 ton of oxygen per day was sufficient since 2013. During the present study period (summer 2015 – 2016) the gas flow rates were reduced. Between June - October 2015 compressed air was supplied at about 105 Nm^3 h^{-1} during 11 h (night) and pure oxygen at 40 Nm^3 h^{-1} for 13 h (day). Finally, in 2016 the supply of pure oxygen was stopped while air was still injected from the end of April until May at 105 Nm^3 h^{-1} over 24 hours until the end of May. From May until September 2016 the aeration was only operated during 11 h at night.

During the phase of highest P concentrations in Lake Hallwil from the 1960’s to the 1990’s *Planktothrix rubescens* was replaced by faster growing algal groups. After 1999, during summer stratification, the scarcity of P along with higher light penetration triggered once again the development of a pronounced layer of *Planktothrix rubescens* in the metalimnion between 6 and 10 m depth which, for the last two decades, made up more than 60% of the lake’s phytoplankton biomass[8].

Supplementary Figures

Station (depth)	Coordinates	
A (45 m)	47°16'45.47"N	8°12'47.39"E
B (40 m)	47°16'48.00"N	8°13'10.00"E
C (20 m)	47°16'50.05"N	8°13'24.23"E
D (2 m)	47°16'49.72"N	8°13'34.79"E
E (20 m)	47°14'59.78"N	8°13'26.09"E
S1 (3 m)	47°16'10.40"N	8°13'45.53"E
S2 (7 m)	47°18'55.71"N	8°12'7.59"E
S3 (23 m)	47°17'35.73"N	8°12'59.61"E
S4 (45 m)	47°16'39.52"N	8°12'53.15"E
M1(45 m)	47°16'43.61"N	8°12'48.93"E
M2(30 m)	47°15'54.36"N	8°13'43.86"E
Diffuser ring (center)	47°17'4.26"N	8°12'45.96"E

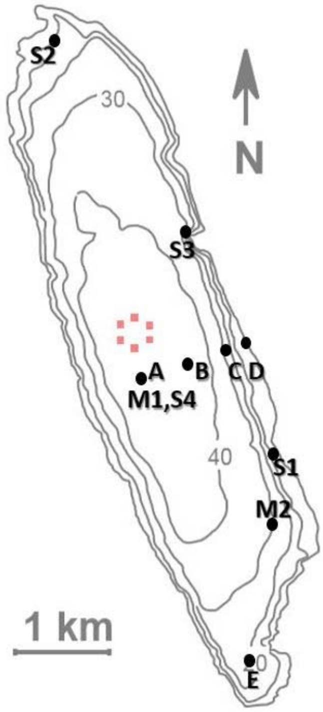

Supplementary Figure 1. Map of the main sampling stations, position of the diffuser and bathymetry (modified after McGinnis et al. [6]).

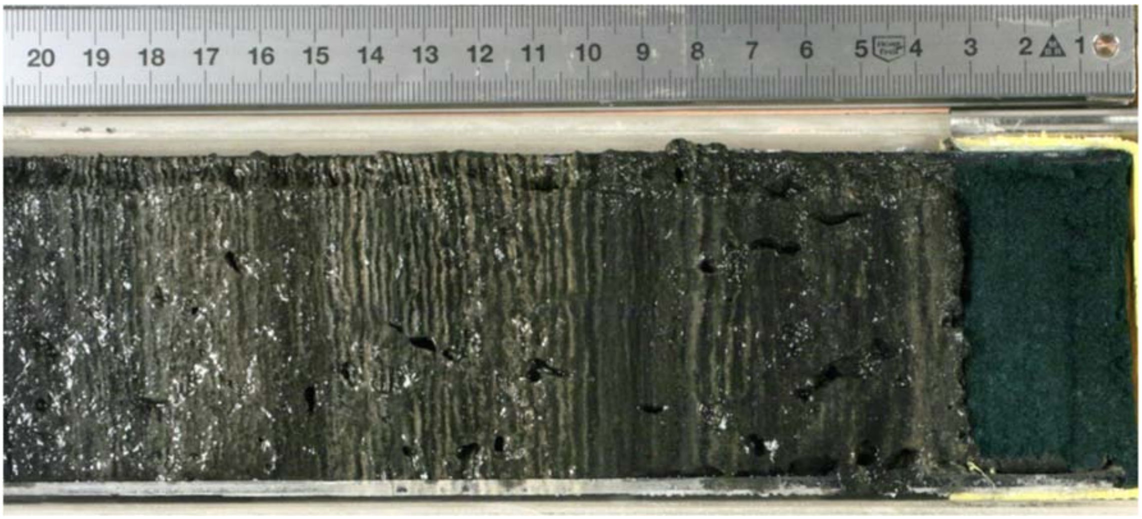

Supplementary Figure 2. Picture of top 20 cm of a core section taken at 45 m depth in Lake Hallwil (St. A) on 12 June 2015. The very well-preserved varves and absence of gas voids indicate that there is not active methane ebullition.

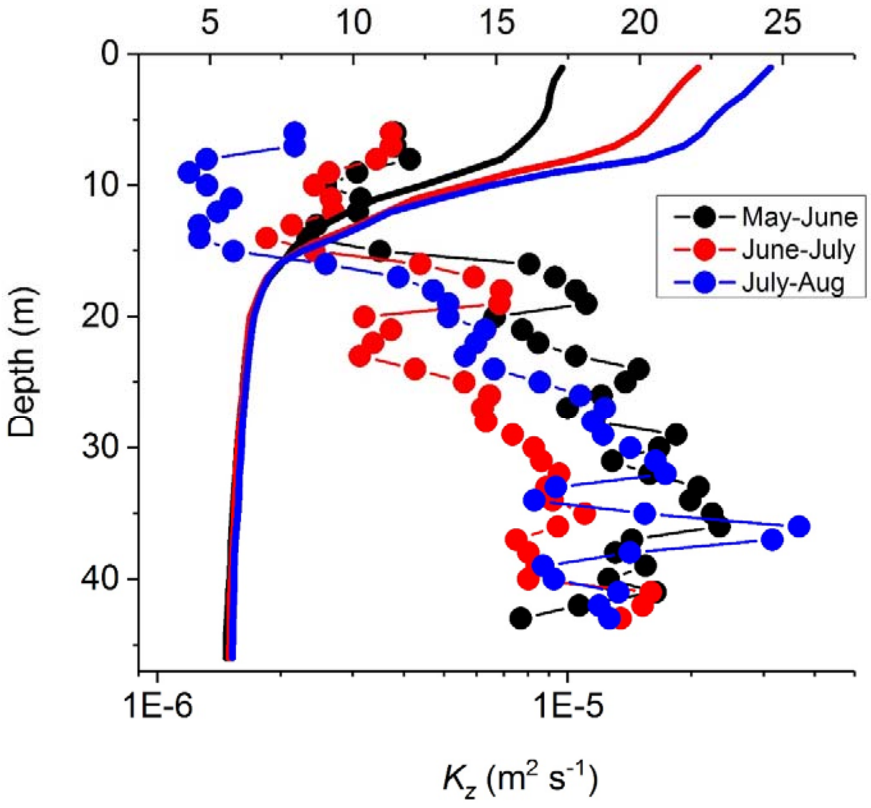

Supplementary Figure 3. Average temperature profiles from CTD casts at St. A for May – June, June – July, July – August 2016 (colored lines) and basin-scale turbulent diffusivities (K_z, circles) computed using the heat budget method from temperatures measured by thermistor chains (Methods) for May – June, June – July, July – August 2016.

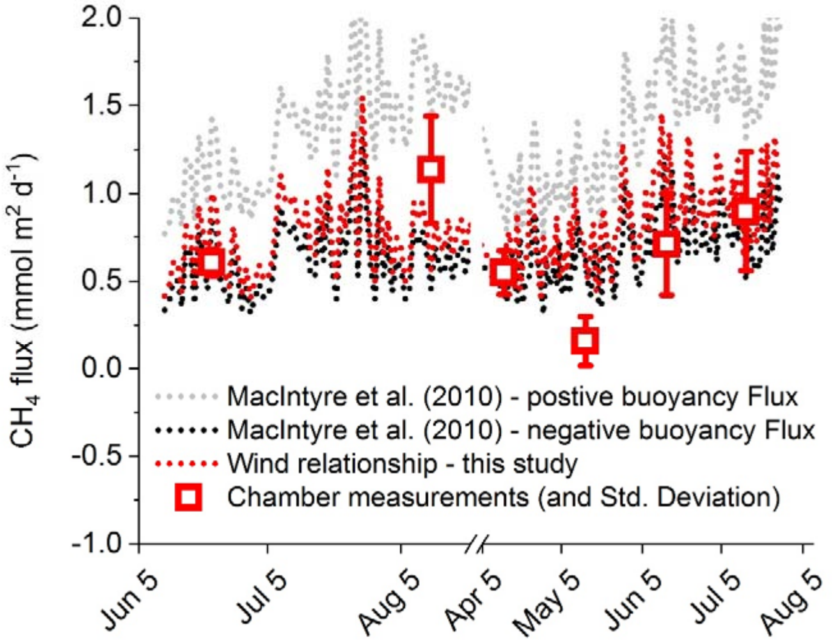

Supplementary Figure 4. Lake Hallwil surface CH_4 fluxes measured with floating chambers (red squares ± 1 SD) and estimated based on different k_{600}, i.e. the wind speed dependent water/air gas transfer coefficient between June 2015 and August 2016 (black dashed line) and coefficient from McIntyre et al. (2010) for positive and negative buoyancy flux. The two chamber measurement series for 12 August 2015 and 15 May 2016 stand out of the wind-based estimates as they were obtained during a particularly windy, cool day and a very warm day with little wind, respectively.

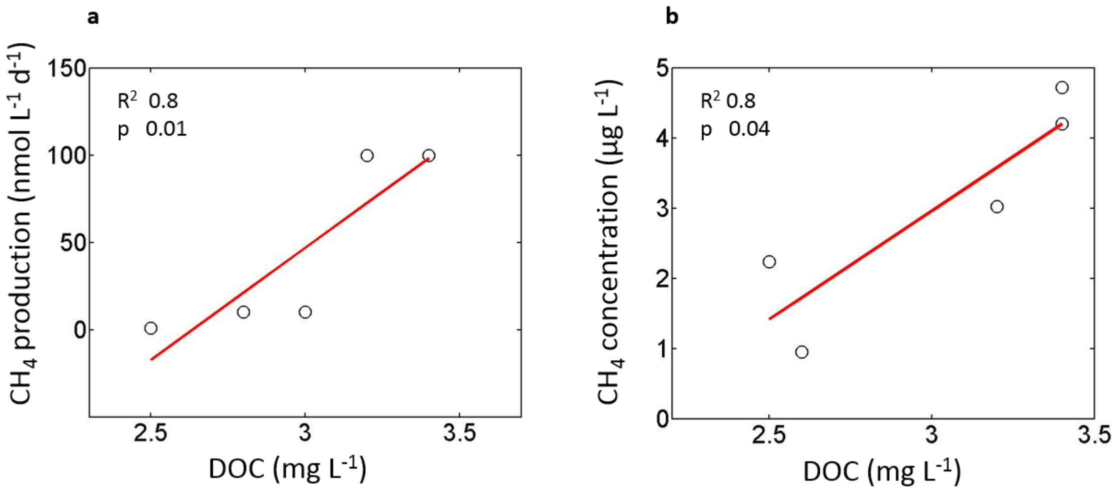

Supplementary Figure 5. a) Linear relationship between top 15 meters P_{net} vs DOC. b) Linear relationship between the temporal (August 2015, October 2015, April 2016, May 2016, August 2016) evolution of the top meter CH_4 concentration vs DOC.

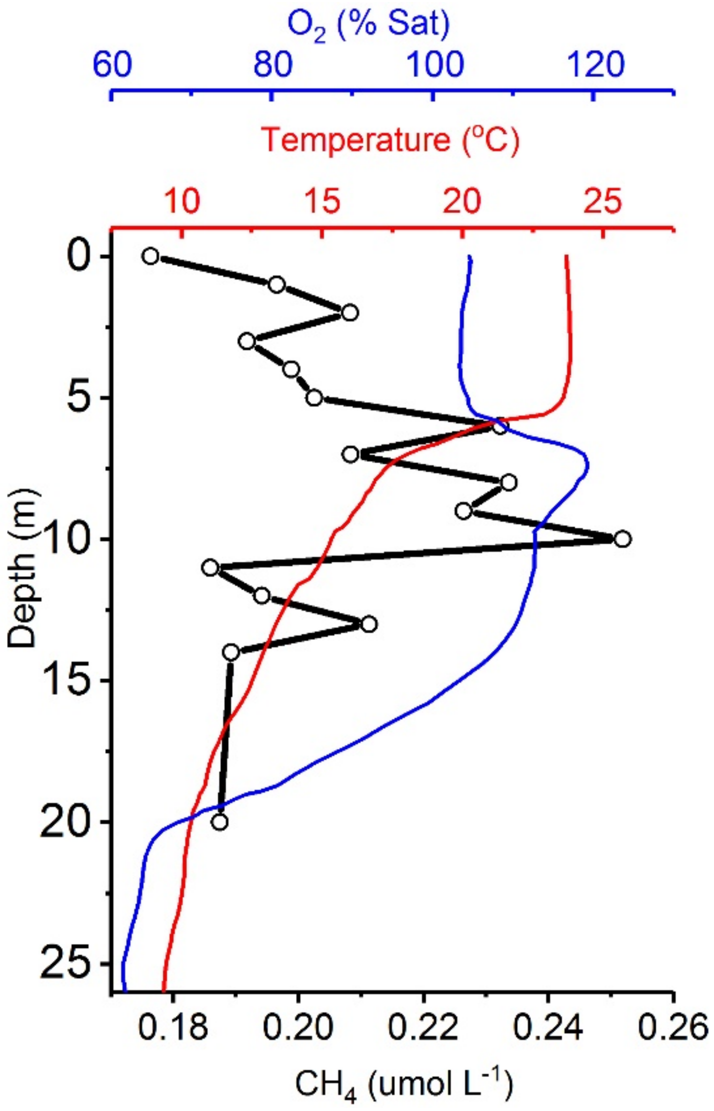

Supplementary Figure 6. Methane, oxygen and temperature profiles for the top 25 m of oligo-mesotrophic Lake Geneva – July 2015.

Supplementary Table 1. Mass balance(s) components range or mean and one standard deviation (1 SD), the number of measurements (n) and date (time range) when measurements were performed.

**Mass balance component**	**Mean/range**	**1 SD (n) unit**	**Measurement**
F_S (Evasion from surface)	0.6	0.3 (28) mmol m^{-2} d^{-1}	April-Aug 2016
$F_{L,sed}$ (Diffusion from littoral sediments)	1.75	0.2 (2) mmol m^{-2} d^{-1}	Sept 2016
$F_{L,eb}$ (Dissolution from littoral ebullition)	1.2	0.8 (8) mmol m^{-2} d^{-1}	Flury *et al.* 2010
F_Z (Diffusion from metalimnion peak)	0.03	0.01 (3) mmol m^{-2} d^{-1}	June-Aug 2016
F_D (CH_4 contributions from aeration system)	0 - 0.014	mmol m^{-2} d^{-1}	
F_R (Input from rivers)	0 - 0.005	nmol L^{-1} d^{-1}	
$P_{gross,m}$ (Production from Eq. 2)	0.01	0.001 nmol L^{-1} d^{-1}	

Supplementary Table 2. Square of correlation coefficient (R^2) and significance (p-value) of water buoyancy frequency (N^2), Chl-*a*, O_2 concentration and turbidity with CH_4 concentration from 0 – 15 meters. Except for July 2015, where CH_4 was strongly correlated to turbidity, during summer season, CH_4 was mostly correlated to water column stability N^2.
* = 0.05 < p > 0.01, ** = 0.01 < p > 0.001, *** = p <0.001.

	**June 2015**		**July 2015**		**Aug 2015**		**June 2016**		**July 2016**		**Aug 2016**	
	**R^2**	**p**	**R^2**	**p**	**R^2**	**p**	**R^2**	**p**	**R^2**	**p**	**R^2**	**p**
CH_4 vs N^2	0.5 **	2E-03	0.3 *	0.04	0.8 ***	1E-06	0.6 ***	4E-05	0.5 **	0.006	0.6 ***	6E-04
CH_4 vs Chl-*a*	<0.1	0.5	0.4 **	8E-03	<0.1	0.4	0.2	0.1	0.2	0.1	0.5 **	1E-03
CH_4 vs O_2	<0.1	0.5	<0.1	0.4	<0.1	0.3	<0.1	0.2	<0.1	0.3	<0.1	0.6
CH_4 vs Turbidity	0.4 *	0.01	0.4 **	3E-03	<0.1	0.2	0.2	0.07	0.2	0.1	<0.1	0.7

Supplementary References

1. Märki, E. & Schmid, M. Der Zustand des Hallwilersees. *Wasser, Energie, Luft* **75** (4), 105–112 (1983).
2. Stöckli, A. Die Sanierung des Hallwilersees macht Fortschritte. *Umwelt Aargau* **1**, 7-10 (1998).
3. Baltzer, P. Besondere Dünge-Vorschriften im Einzugsgebiet des Hallwilersees. *Umwelt Aargau* **58**, 9–12 (2012).
4. Stöckli, A. & Schmid, M. Die Sanierung des Hallwilersees: Erste Erfahrungen mit Zwangszirkulation und Tiefenwasserbelüftung, *Wasser Energie, Luft* **79** (7/8), 143–149 (1987).
5. Bürgi, H. R. & Jolidon, C. 10 Jahre Seesanierung Hallwilersee: die Reaktion des Planktons. *Wasser, Energie, Luft* **90** (5/6), 109–116 (1998).
6. McGinnis, D. F., Lorke, A., Wüest, A., Stöckli, A. & Little, J. C. Interaction between a bubble plume and the near field in a stratified lake. *Water Resour. Res*. **40**, 1–11 (2004).
7. Stöckli A. Hallwilersee – nachhaltige Gesundung sicherstellen. *Umwelt Aargau* **69**, 23–28 (2015).
8. Stöckli, A. Das Plankton zeigt die Gesundung des Hallwilersees. *Umwelt Aargau* **58**, 13–20 (2012).
